# Offline Digital Education for Medical Students: Systematic Review and Meta-Analysis by the Digital Health Education Collaboration

**DOI:** 10.2196/13165

**Published:** 2019-03-25

**Authors:** Bhone Myint Kyaw, Pawel Posadzki, Gerard Dunleavy, Monika Semwal, Ushashree Divakar, Vasilis Hervatis, Lorainne Tudor Car

**Affiliations:** 1 Family Medicine and Primary Care Lee Kong Chian School of Medicine Nanyang Technological University Singapore Singapore; 2 Centre for Population Health Sciences (CePHaS) Lee Kong Chian School of Medicine Nanyang Technological University Singapore Singapore; 3 Department of Learning, Informatics, Management and Ethics Karolinska Institutet Stockholm Sweden; 4 Department of Primary Care and Public Health School of Public Health Imperial College London London United Kingdom

**Keywords:** medical education, systematic review, meta-analysis, randomized controlled trials, students, medical

## Abstract

**Background:**

Medical schools in low- and middle-income countries are facing a shortage of staff, limited infrastructure, and restricted access to fast and reliable internet. Offline digital education may be an alternative solution for these issues, allowing medical students to learn at their own time and pace, without the need for a network connection.

**Objective:**

The primary objective of this systematic review was to assess the effectiveness of offline digital education compared with traditional learning or a different form of offline digital education such as CD-ROM or PowerPoint presentations in improving knowledge, skills, attitudes, and satisfaction of medical students. The secondary objective was to assess the cost-effectiveness of offline digital education, changes in its accessibility or availability, and its unintended/adverse effects on students.

**Methods:**

We carried out a systematic review of the literature by following the Cochrane methodology. We searched seven major electronic databases from January 1990 to August 2017 for randomized controlled trials (RCTs) or cluster RCTs. Two authors independently screened studies, extracted data, and assessed the risk of bias. We assessed the quality of evidence using the Grading of Recommendations, Assessment, Development, and Evaluations criteria.

**Results:**

We included 36 studies with 3325 medical students, of which 33 were RCTs and three were cluster RCTs. The interventions consisted of software programs, CD-ROMs, PowerPoint presentations, computer-based videos, and other computer-based interventions. The pooled estimate of 19 studies (1717 participants) showed no significant difference between offline digital education and traditional learning groups in terms of students’ postintervention knowledge scores (standardized mean difference=0.11, 95% CI –0.11 to 0.32; small effect size; low-quality evidence). Meta-analysis of four studies found that, compared with traditional learning, offline digital education improved medical students’ postintervention skills (standardized mean difference=1.05, 95% CI 0.15-1.95; large effect size; low-quality evidence). We are uncertain about the effects of offline digital education on students’ attitudes and satisfaction due to missing or incomplete outcome data. Only four studies estimated the costs of offline digital education, and none reported changes in accessibility or availability of such education or in the adverse effects. The risk of bias was predominantly high in more than half of the included studies. The overall quality of the evidence was low (for knowledge, skills, attitudes, and satisfaction) due to the study limitations and inconsistency across the studies.

**Conclusions:**

Our findings suggest that offline digital education is as effective as traditional learning in terms of medical students’ knowledge and may be more effective than traditional learning in terms of medical students’ skills. However, there is a need to further investigate students’ attitudes and satisfaction with offline digital education as well as its cost-effectiveness, changes in its accessibility or availability, and any resulting unintended/adverse effects.

## Introduction

There is a global shortage of 2.6 million medical doctors according to the World Health Organization [1]. In low- and middle-income countries, this shortage is further exacerbated by migrations, inadequacy of training programs, and poor infrastructure including limited access to fast and reliable internet connection [2-6]. Additionally, the content, structure, and delivery mode of medical curricula in these countries are often inadequate to equip medical students with the required knowledge, skills, and experience needed to meet their populations’ evolving health care needs [7]. To tackle these multifaceted and intertwined problems, complex measures need to be taken to increase not only the number of medical doctors, but also the quality and relevance of their training [8]. Offline digital education offers a potential solution to overcome these problems.

Offline digital education, herein also referred to as computer-based learning or computer-assisted instruction, was one of the first forms of digital education, used before the internet became available on a global scale [9,10]. Unlike online digital education, offline digital education is independent of the internet or a local area network connection. Offline digital education can be delivered through CD-ROM, digital versatile disc (DVD)-ROM, external hard discs, universal serial bus (USB) memory sticks, or different software packages [11]. Offline digital education offers potential benefits over traditional modes of learning, including self-paced directed learning, stimulation of various senses (eg, with visual and spatial components) [12,13], and the ability to represent content in a variety of media (eg, text, sound, and motion) [14]. The educational content of the interventions is highly adaptable to the learners’ needs, with the potential to be reviewed, repeated, and resumed at will. The interventions offer improved accessibility and flexibility and transcend the geographical, temporal, and financial boundaries that medical students may face. By reducing the costs of transportation or renting out classrooms and by freeing up the time of medical curriculum providers [14-16], the interventions may potentially offer substantial monetary savings.

A number of randomized control trials (RCTs) have evaluated the effectiveness of offline digital education in improving learning outcomes of medical students. Some of these trials were further evaluated in systematic reviews [13,17], but the findings were inconclusive. The primary objective of this systematic review was to evaluate the effectiveness of offline digital education compared with traditional learning or different forms of offline digital education in improving medical students’ knowledge, skills, attitudes, and satisfaction. The secondary objective was to assess the economic impact of offline digital education, changes in its accessibility or availability, and its unintended/adverse effects.

## Methods

### Protocol

For this systematic review, we adhered to the published protocol [18]. The methodology has been described in detail by the Digital Health Education Collaboration [19]. The Digital Health Education collaboration is a global initiative focused on evaluating the effectiveness of digital health professions education through a series of methodologically robust systematic reviews.

### Search Strategy and Data Sources

#### Electronic Searches

We developed a comprehensive search strategy for MEDLINE (Ovid; see Multimedia Appendix 1 for MEDLINE [Ovid] search strategy), Embase (Elsevier), Cochrane Central Register of Controlled Trials (CENTRAL) (Wiley), PsycINFO (Ovid), Educational Research Information Centre (Ovid), Cumulative Index to Nursing and Allied Health Literature (Ebsco), and Web of Science Core Collection (Thomson Reuters). Databases were searched from January 1990 to August 2017. We selected 1990 as the starting year for our search because prior to this year, the use of computers was limited to very basic tasks. There were no language restrictions. We searched the reference lists of all the studies that we deemed eligible for inclusion in our review and the relevant systematic reviews. We also searched the International Clinical Trials Registry Platform Search Portal and metaRegister of Controlled Trials to identify unpublished trials.

We developed a common, comprehensive search strategy for a series of systematic reviews focusing on different types of digital education (ie, offline digital education, online digital education, and mobile learning) for preregistration as well as postregistration health care professionals. We retrieved 30,532 records from different bibliographic databases initially. In this review, we only included studies focusing on the effectiveness of offline digital education in medical students’ education, and the findings on other types of digital education (such as virtual reality and mobile learning) within health professions education were reported separately [20-26].

For the purpose of this review, offline digital education can be defined as offline and stand-alone computer-based or computer-assisted learning where internet or intranet connection is not required for the learning activities. Traditional learning can be defined as learning via traditional forms of education such as paper- or text book–based learning and didactic or face-to-face-lecture. Blended digital education can be defined as any intervention that involves the combined use of offline digital education and traditional learning.

#### Inclusion Criteria

We included RCTs and cluster RCTs (cRCTs). Crossover trials were excluded due to a high likelihood of carry-over effect. We included studies with medical students enrolled in a preregistration, university degree program. Participants were not excluded based on age, gender, or any other sociodemographic characteristic.

We included studies in which offline digital education was used to deliver the learning content of the course. This included studies focused solely on offline digital education, or where offline digital education was part of a complex, multicomponent intervention. The main tasks of the learning activities were performed on a personal computer or laptop (with a hard keyboard). The delivery channel of the computer-based intervention was typically accessed via software programs, CD-ROM, DVD, hard disc, or USB memory stick. The focus was mainly on the learning activities that do not have to rely on any internet or online connection. Interventions where the internet connection was essential to provide learning content were excluded from this review.

We included the control groups that comprised traditional learning or traditional face-to-face learning such as lectures or discussions or text- or textbook-based learning as well as other offline digital education. We included studies that compared offline digital education or blended learning to traditional learning or a different form of offline digital education such as CD-ROM or PowerPoint presentations.

Learning outcomes were chosen based on the literature and relevance for medical students’ education [27]. Eligible studies had to report at least one of the specified primary or secondary outcomes. Primary outcomes (measured using any validated or nonvalidated instruments) were medical students’ knowledge scores (postintervention), medical students’ cognitive skills (postintervention), medical students’ postintervention attitudes toward the interventions or new clinical knowledge, and medical students’ postintervention satisfaction with the interventions. Secondary outcomes included the economic impact of offline digital education (eg, cost-effectiveness, implementation cost, and return on investment), changes in its accessibility or availability, and any resulting adverse effects.

### Data Collection and Analysis

#### Selection of Studies

The search results from different electronic databases were combined in a single Endnote (X.8.2) library, and duplicate records were removed [28]. Four review authors (BK, GD, MS, and UD) independently screened titles and abstracts of all the records to identify potentially eligible studies. We retrieved full-text copies of the articles deemed potentially relevant. Finally, two reviewers (BK and GD) independently assessed the full-text versions of the retrieved articles against the eligibility criteria. Any disagreements were resolved through discussion between the two reviewers, with a third review author (PP) acting as an arbiter, when necessary.

#### Data Extraction and Management

Five reviewers (BK, GD, MS, UD, and VH) independently extracted relevant characteristics related to participants, intervention, comparators, outcome measures, and results from all the included studies using a standard data-collection form. Any disagreements between the reviewers were resolved by discussion. We contacted the study authors for any missing information, particularly information required to judge the risk of bias.

#### Assessment of Risk of Bias in Included Studies

Four reviewers (BK, GD, MS, and UD) independently assessed the methodological risk of bias of included studies using the Cochrane methodology [29]. The following individual risk-of-bias domains were assessed in the included RCTs: random sequence generation, allocation concealment, blinding (outcome assessment), completeness of outcome data (attrition bias), selective outcome reporting (relevant outcomes reported), and other sources of bias (baseline imbalances).

For cRCTs, we assessed the risk of the following additional domains: recruitment bias, baseline imbalance, loss of clusters, incorrect analysis, and comparability with individually randomized trials recommended by Puffer et al [30]. Judgements concerning the risk of bias for each study were scored as high, low, or unclear. We incorporated the results of the risk-of-bias assessment into the review using a graph and a narrative summary. We also assessed publication bias using a funnel plot for comparisons with at least 10 studies.

#### Measures of Treatment Effect

For continuous outcomes, we reported mean postintervention scores and SD in each intervention group along with the number of participants and *P* values. We reported mean postintervention outcome data to ensure consistency across the included studies, as this was the most commonly reported form of findings. We presented outcomes using postintervention standardized mean difference (SMD) and interpreted the effect size using the Cohen rule of thumb (ie, with 0.2 representing a small effect, 0.5 representing a moderate effect, and 0.8 representing a large effect) [29,31]. For dichotomous outcomes, we calculated the risk ratio and 95% CIs. If studies had multiple arms, we compared the most active intervention arm to the least active control arm and assessed the difference in postintervention outcomes. We used the standard method recommended by Higgins et al to convert the results [29].

#### Data Synthesis

For meta-analysis, we used a random-effects model. For studies with the same continuous outcome measures, SMDs (for different scales) between groups, along with the 95% CIs, were estimated using Review Manager 5.3 [32]. In the analysis of continuous outcomes and cRCTs, we used the inverse variance method. We displayed the results of the meta-analyses in forest plots that provided effect estimates and 95% CIs for each individual study as well as a pooled effect estimate and 95% CI. For every step in the data analysis, we adhered to the statistical guidelines described by Higgins et al in 2011 [29].

**Table 1:** Summary of findings table: Effects of offline digital education on knowledge, skills, attitudes, and satisfaction. Patient or population: medical students, Settings: university or hospital, Intervention: offline digital education, Comparison: offline digital education versus traditional learning.

Outcomes	Illustrative comparative risks (95% CI)	Number of participants (number of studies)	Quality of the evidence (GRADE^a^)	Comments
Knowledge: Assessed with multiple-choice questions, questionnaires, essays, quizzes, and practical section (from postintervention to 11-22 months of follow-up)	The mean knowledge score in offline digital education groups was 0.11 SD higher (–0.11 lower to 0.32 higher)	1717 (19)	Low^b,c,d^	The results from seven studies (689 participants) were not added to the meta-analysis due to incomplete or incomparable outcome data. These studies reported mixed findings: four studies (331 participants) favored offline digital education group, two studies reported no difference (289 participants), and one study favored the traditional learning group (69 participants).
Skills: Assessed with checklists, Likert-type scales, and questionnaires, (from postintervention to 1-10 months of follow-up)	The mean skills score in the offline digital education groups was 0.5 SD higher (0.25 higher to 0.75 higher)	415 (4)	Low^b,c,d^	The results of two studies (190 participants) were not added to the meta-analysis due to incomplete outcome data. One study (121 participants) favored offline digital education group. The other study (69 participants) reported no difference between the groups immediately postintervention and favored the offline digital education group at 1-month of follow-up.
Attitude: Assessed with Likert scale, questionnaires, and surveys (from postintervention to 5 weeks of follow-up)	Not estimable	493 (5)	Low^b,c,d^	One study (54 participants) reported higher postintervention attitude scores in offline digital education compared to traditional learning. We were uncertain about the effect of four studies (439 participants) due to incomplete outcome data.
Satisfaction: Assessed with Likert scales, questionnaires, and surveys (postintervention)	Not estimable	1442 (15)	Low^b,c,d^	Two studies (144 participants) favored traditional learning and two studies (103 participants) reported little or no difference between the groups. We were uncertain about the effect of 11 studies (1195 participants) due to incomplete outcome data.

^a^GRADE: Grading of Recommendations, Assessment, Development, and Evaluations.

^b^Low quality: Further research is very likely to have an important impact on our confidence in the estimate of effect and is likely to change the estimate.

^c^Rated down by one level for study limitations. The risk of bias was unclear for sequence generation and allocation concealment in majority of the studies.

^d^Rated down by one level for inconsistency. The heterogeneity is high with large variations in effect and lack of overlap among CIs.

We synthesized the findings from the included studies by the type of comparison: offline digital education (including PowerPoint and CD-ROM) versus traditional learning, offline digital education versus a different form of offline digital education, and blended learning versus traditional learning. Two authors (BK and GD) used the Grading of Recommendations, Assessment, Development, and Evaluations criteria to assess the quality of the evidence [33]. We considered the following criteria to evaluate the quality of the evidence, downgrading the quality where appropriate: limitations of studies (risk of bias), inconsistency of results (heterogeneity), indirectness of the evidence, imprecision (sample size and effect estimate), and publication bias. We prepared a summary of findings table [33] to present the results (Table 1). Where a meta-analysis was unfeasible, we presented the results in a narrative format, such as that used by Chan et al [34].

## Results

### Results of the Search

**Figure 1:**
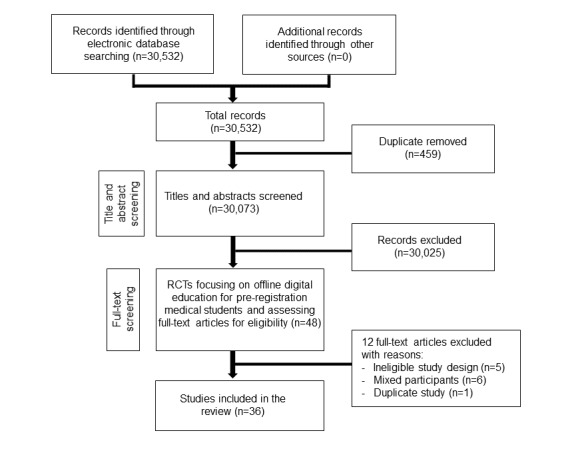
Preferred Reporting Items for Systematic Reviews and Meta-Analyses flow diagram. RCT: randomized controlled trials.

Our search strategy retrieved 30,532 unique references (Figure 1).

### Included Studies

**Figure 2:**
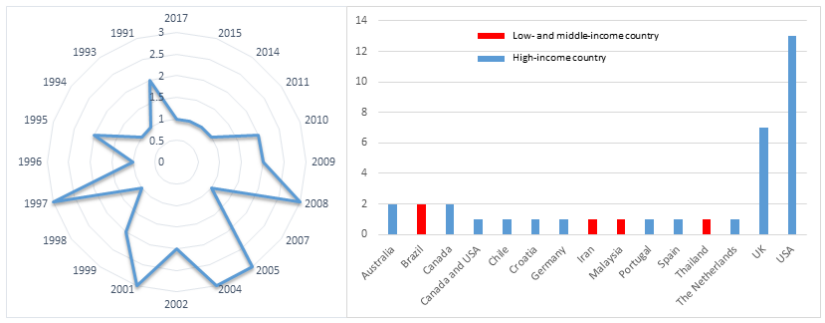
Number of publication(s) on offline digital education in relation to their year of publication and country of origin.

We included 36 studies from 35 reports involving 3325 participants [10,35-68] (Table 2), of which 33 studies were RCTs and the remaining three studies (from two reports) were cRCTs [56,68]. One study [68] reported the results of two cRCTs and we reported these results separately. Thirty-two studies (89%) were published before 2010, and only four studies (11%) were published after 2010 (Figure 2) [39,48,55,66].

The number of participants across the studies varied from 20 [35] to 241 [68], while individual studies focused on different areas of medical education. For the intervention groups, 20 studies used software programs [10,42,43,45,48-51,53,54, 56,58-62,64-67], nine used CD-ROMs [35,36,40,44, 46,55,57,68], four used PowerPoint presentations [37,38,41,63], two did not specify the type of intervention [47,52], and one used a computer-based video [39]. The duration of the interventions ranged from 10 minutes [39] to 3 weeks [41]. Four studies did not report the duration of the intervention [47,48,56,63]. The frequency of the intervention ranged from one [35,38-40,48,49,51,52,55,57-61,64,65,67-68] to six [50], and the intensity ranged from 10 minutes [39] to 11.1 hours [53]. Nine studies provided instructions on how to use the software [39,42,44,46,50,53,65,68]. Eight studies reported security arrangements [36,37,43,45,49,50,53,58]. For the control groups, 30 studies used traditional methods of learning such as face-to-face lectures, paper- or text book–based learning resources, laboratory courses, practical workshops, or small group tutorials [10,36-42,44,45,47-57,59-65,67,68]. Five studies used different forms of offline digital education as the controls [35,43,46,58,66]. One study compared blended learning (computer-assisted learning in addition to traditional learning) and traditional learning alone [44]. More information on the types of interventions is provided in Multimedia Appendix 2.

**Table 2:** Characteristics of the included studies.

Study, design, and country	Population (n), (medical student year)	Field of study	Outcomes
Ackermann et al 2010 [35], RCT^b^, Germany	20 (not specified)	Surgery (orthopedic surgery)	Skill
Amesse 2008 [36], RCT, United States	36 (third year)	Radiology	Knowledge
Armstrong et al 2009 [37], RCT, United Kingdom	21 (fourth year)	Arterial blood gas interpretation	Knowledge and satisfaction
Carrero et al 2009 [38], RCT, Spain	68 (third year)	Basic life support algorithms	Knowledge
Cheng et al 2017 [39], RCT, United States	41 (second, third and fourth year)	Orthopedics	Skill
Davis et al 2008 [40], RCT, United Kingdom	229 (first year)	Evidence-based medicine	Knowledge and attitude
de Jong et al 2010 [41], RCT, The Netherlands	107 (third year)	Musculoskeletal problems	Knowledge and satisfaction
Desch et al 1991 [42], RCT, United States	78 (third year)	Pediatrics (neonatal management)	Knowledge, satisfaction, and cost
Devitt and Palmer 1999 [43], RCT, Australia	90 (second year)	Anatomy and physiology	Knowledge
Elves et al 1997 [44], RCT, United Kingdom	26 (third year)	Urology	Knowledge and satisfaction
Fasce et al 1995 [45], RCT, Chile	100 (fourth year)	Medicine (hypertension)	Knowledge, attitude, and satisfaction
Finley et al 1998 [46], RCT, Canada	40 (second year)	Medicine (auscultation of heart)	Knowledge and satisfaction
Gelb 2001 [47], RCT, United States	107 (not specified)	Anatomy	Knowledge and satisfaction
Green and Levi 2011 [48], RCT, United States	121 (second year)	Advanced care planning	Knowledge, skill, and satisfaction
Hilger et al 1996 [49], RCT, United States	77 (third year)	Medicine (pharyngitis)	Knowledge and attitude
Hudson 2004 [10], RCT, Australia	100 (third year)	Neuroanatomy and neurophysiology	Knowledge
Holt et al 2001 [50], RCT, United Kingdom	185 (first year)	Endocrinology	Knowledge, satisfaction, and cost
Lee et al 1997 [51], RCT, United States	82 (second year)	Biochemistry/acid-base problem solving	Knowledge and satisfaction
MacFadyen et al 1993 [52], RCT, Canada	54 (fourth year)	Clinical pharmacology	Knowledge and attitude
Mangione et al 1991 [53], RCT, United States	35 (third year)	Auscultation of the heart	Knowledge and attitude
McDonough and Marks 2002 [54], RCT, United Kingdom	37 (third year)	Psychiatry	Knowledge and satisfaction
Mojtahedzadeh et al 2014 [55], RCT, Iran	61 (third year)	Physiology of hematology and oncology	Knowledge and satisfaction
Nola et al 2005 [56], cRCT^c^, Croatia	225 (not specified)	Pathology	Knowledge
Perfeito et al 2008 [57], RCT, Brazil	35 (fourth year)	Surgery	Knowledge and satisfaction
Pusic et al 2007 [58], RCT, Canada and United States	152 (final year)	Radiology	Knowledge and satisfaction
Ram 1997 [59], RCT, Malaysia	64 (final year)	Cardiology	Knowledge
Santer et al 1995 [60], RCT, United States	179 (third and fourth year)	Pediatrics	Knowledge and satisfaction
Seabra et al 2004 [61], RCT, Brazil	60 (second and third year)	Urology	Knowledge and satisfaction
Shomaker et al 2002 [62], RCT, United States	94 (second year)	Parasitology	Knowledge and satisfaction
Solomon et al 2004 [63], RCT, United States	29 (third year)	Learning concepts (digital and live lecture formats)	Knowledge
Stanford et al 1994 [64], RCT, United States	175 (first year)	Anatomy (cardiac anatomy)	Knowledge and satisfaction
Summers et al 1999 [65], RCT, United States	69 (first year)	Surgery	Knowledge and skill
Taveira-Gomes et al 2015 [66], RCT, Portugal	96 (fourth and fifth year)	Cellular biology	Knowledge
Vichitvejpaisal et al 2001 [67], RCT, Thailand	80 (third year)	Arterial blood gas interpretation	Knowledge
Vivekananda-Schmidt et al 2005 [68], cRCT, United Kingdom (Newcastle)^c^	241 (third year)	Orthopedics (musculoskeletal examination skills)	Skill and cost
Vivekananda-Schmidt et al 2005 [68], cRCT, United Kingdom (London)^c^	113 (third year)	Orthopedics (musculoskeletal examination skills)	Skill and cost

^a^RCT: randomized controlled trial.

^b^cRCT: cluster randomized controlled trial.

^c^This study reported its results from two separate cRCTs. We analyzed data from the two cRCTs separately.

#### Knowledge

##### Overview

Knowledge was assessed as the primary outcome in 32 studies [10,36-38,40-67], and majority of the studies (69%) used multiple-choice questions or questionnaires to measure the outcome. Twenty-nine studies assessed knowledge using nonvalidated instruments [36-38,40-49,51-57,59-67], while three studies [10,50,58] used validated instruments such as multiple-choice questions, questionnaires, and tests. Twenty-six studies assessed postintervention knowledge scores, while six studies assessed both short-term and long-term knowledge retention, ranging from 1 week to 22 months of follow-up [52,60,62,65-67] (Multimedia Appendix 3).

##### Offline Digital Education Versus Traditional Learning

**Figure 3:**
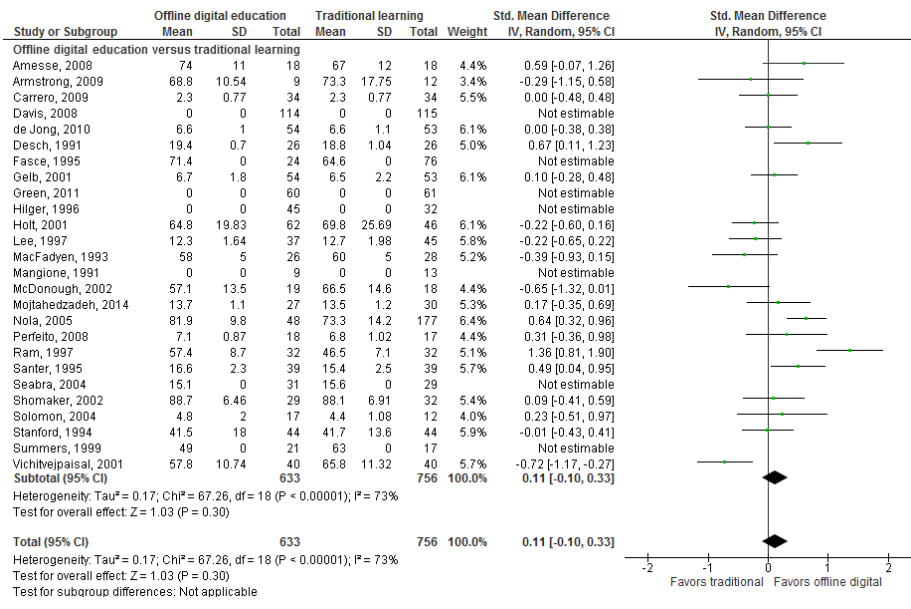
Forest plot of studies comparing offline digital education with traditional, postintervention knowledge outcome. IV=interval variable; random=random effect model.

Meta-analysis of 19 studies showed that there was no significant difference between offline digital education and traditional learning in postintervention knowledge scores (SMD 0.11, 95% CI –0.11 to 0.32; 1717 participants; small effect size, low-quality evidence; Figure 3). There was a substantial amount of heterogeneity in the pooled analyses (*I*^2^=73%). The remaining seven studies were not pooled due to incomplete or incomparable outcome data [40,45,48,49,53,61,65] and reported mixed findings. Four studies reported a significant difference in postintervention knowledge scores in favor of offline digital education (331 participants) [45,48,49,53]. Two studies reported no significant difference between the interventions (289 participants) [40,61], and one study (69 participants) reported a significant difference in postintervention knowledge scores in favor of traditional learning [65]. Taken together, these findings suggest that offline digital education had similar effects as traditional learning on medical students’ postintervention knowledge scores.

##### Blended Learning Versus Traditional Learning

One study (26 participants) assessed postintervention knowledge scores in blended learning (offline digital education plus traditional learning) versus traditional learning alone and reported a significant difference in favor of blended learning (SMD 0.81, 95% CI 0.01-1.62; large effect size) [44].

##### Offline Digital Education Versus Offline Digital Education

Five studies (478 participants) compared one form of offline digital education (eg, CD-ROM and software programs) to another form of offline digital education (eg, CD-ROM, software programs, or computer-assisted learning programs) [10,43,46,58,66].

Devitt et al reported higher knowledge scores in the computer-based group than a free-text entry program (SMD 1.62, 95% CI 0.93-2.31; large effect size) at 2 weeks postintervention [43]. Taveira-Gomes et al also reported higher postintervention knowledge scores in the computer-based software group (ie, the use of *flashcards-based learning materials on cellular structure*) compared to a computer-based method alone (ie, without the use of flashcards; SMD 2.17, 95% CI 1.67-2.67, large effect size) [66]. Pusic et al reported that the effectiveness of a simple linear computer program was equivalent to that of a more interactive, branched version of the program in terms of postintervention knowledge scores (SMD 0, 95% CI –0.33 to 0.33; small effect size) [58]. The effect of two studies was uncertain due to incomplete outcome data [10,46]. Overall, the findings were mixed and inconclusive.

#### Skills

##### Overview

Six studies from five reports (605 participants) assessed skills as a primary outcome [35,39,48,65,68]. Three studies used validated instruments to measure the outcome such as an Objective Structured Clinical Examination [68] and a checklist rating form [65]. The remaining three studies used nonvalidated instruments such as questionnaires [35], a six-point checklist scale [39], and self-assessment [48]. Four studies [35,39,48,68] assessed postintervention skills scores, while two studies [65,68] assessed both short-term and long-term skill retention, ranging from 1 month to 10 months of follow-up (Multimedia Appendix 3).

##### Offline Digital Education Versus Traditional Learning

**Figure 4:**
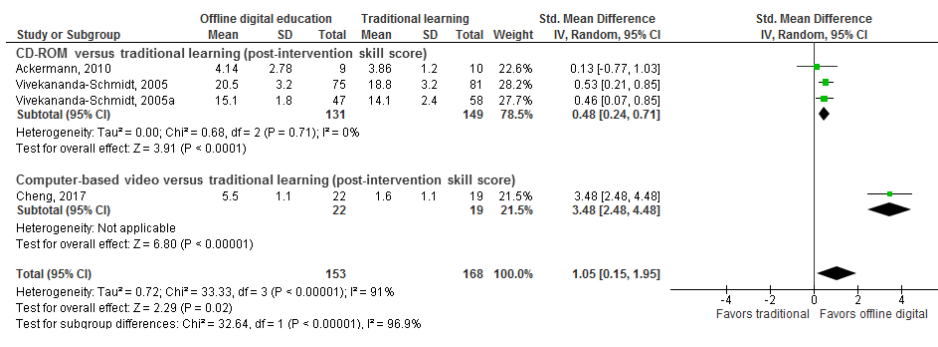
Forest plot of studies comparing offline digital education with traditional, postintervention skill outcome. IV=interval variable; random=random effect model. Vivekananda-Schmidt, 2005 was conducted in Newcastle and Vivekananda-Schmidt, 2005a was conducted in London.

Meta-analysis of four studies showed that, compared with traditional learning, offline digital education improved medical students’ postintervention skill scores (SMD 1.05, 95% CI 0.15-1.95; *I*^*2*^=91%; large effect size; low-quality evidence; Figure 4). There was, however, a considerable amount of heterogeneity in the pooled analyses (*I*^*2*^=91%).

The results of two studies were not pooled due to incomplete outcome data [48,65]. These studies also reported higher postintervention skill scores with offline digital education than with traditional learning [48,65]. Taken together, these results suggest that offline digital education may improve postintervention skill scores compared to traditional learning.

#### Attitude

Five studies (493 participants) reported students’ postintervention attitude toward the intervention or new clinical knowledge [40,45,49,52,53]. Attitudes were measured using Likert-based questionnaires [40,49,52], surveys [45], and the Computer Anxiety Index [53]. None of the studies used validated tools to measure the outcome. MacFadyen et al reported higher postintervention attitude scores in the offline digital education group than in the traditional learning group (SMD 2.71, 95% CI 1.96-3.47; large effect size) [52]. One study reported higher attitude scores in the intervention group than in the traditional learning group (89% vs 47%) [45]. Two studies did not report numerical data for either of the study groups [40,49], while one study assessed participants’ postintervention attitudes in the intervention group only [53]; hence, we were unable to judge the effect of these three interventions due to missing outcome data [40,45,49,53]. Taken together, the overall effect of the interventions seems uncertain due to the lack of outcome data in most of the included studies.

#### Satisfaction

Eighteen studies (1660 participants) assessed postintervention satisfaction [37,41,42,44-48,50,51,54,55,57,58,60-62,64]. Nine studies used Likert-type rating scales [42,46,48,51, 54,55,58,61,62], eight studies used questionnaires [37,41,44,47,50,57,60,64], and one study [45] used a survey to assess participants’ postintervention satisfaction. None of the studies used validated tools to measure the outcome.

Fifteen studies comparing offline digital education with traditional learning assessed satisfaction. Two studies [41,54] reported higher postintervention satisfaction scores in the traditional learning group than in offline digital education (risk ratio=0.46, 95% CI 0.30-0.69; small effect size; SMD –1.33, 95% CI –2.05 to –0.61, large effect size). Two other studies reported no significant difference in students’ postintervention satisfaction between the groups [37,51]. The remaining 11 studies reported incomplete or incomparable outcome data [42,45,47,48,50,55,57,60-62,64]. Overall, we were uncertain about the effects of offline digital education on students’ satisfaction scores, when compared with traditional learning.

Three studies comparing different forms of offline digital education and blended learning to traditional learning also assessed satisfaction. However, we were unable to judge the overall effect of the intervention in the three studies due to missing or incomparable outcome data [44,46,58].

### Secondary Outcomes

Four studies (617 participants) reported the cost of the offline digital education [42,50,68]. However, none of the included studies compared costs between the intervention and control groups.

Desch et al reported that the authoring system (computer-assisted instructional software program) costs US $600. Additionally, the study used US $1500 to hire a student to develop the program [42]. The microcomputers used by the students in the study by Desch et al were within a large microcomputer area in the medical library and were used for multiple purposes. Holt et al reported that the total cost of the equipment specially needed to set up the computer-assisted learning course (including slide and document scanners, sound recording, a laptop, and software) was approximately £3000 (~US $4530) [50]. Two studies reported that the cost of designing a virtual rheumatology CD was £11,740 (US $22,045) [68].

No studies reported adverse or unintended effects of the interventions or changes in the accessibility or availability of digital offline education.

**Figure 5:**
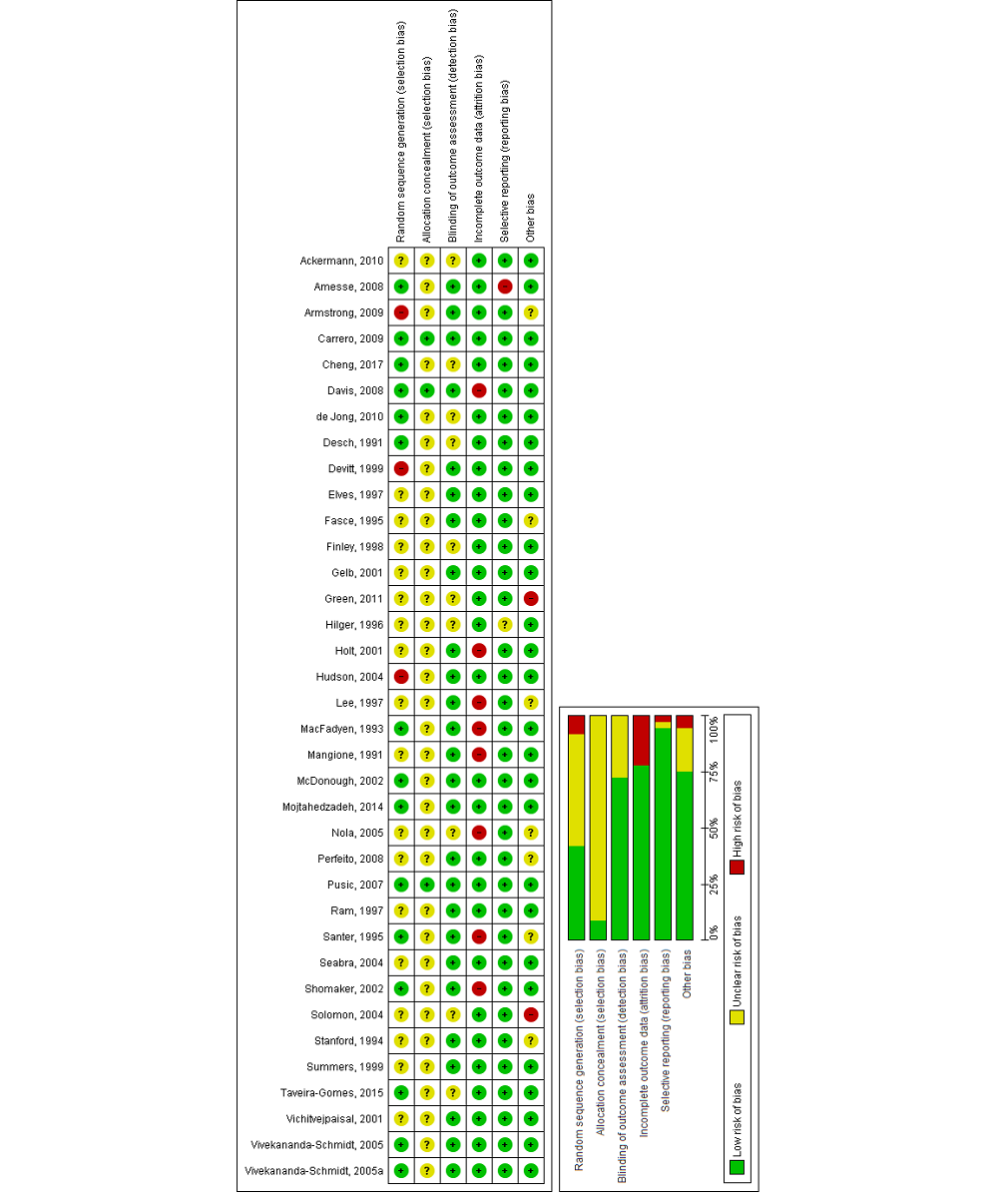
Risk of bias summary: review authors' judgements about each risk of bias item across all included studies.

### Risk of Bias in Included Studies

**Figure 6:**
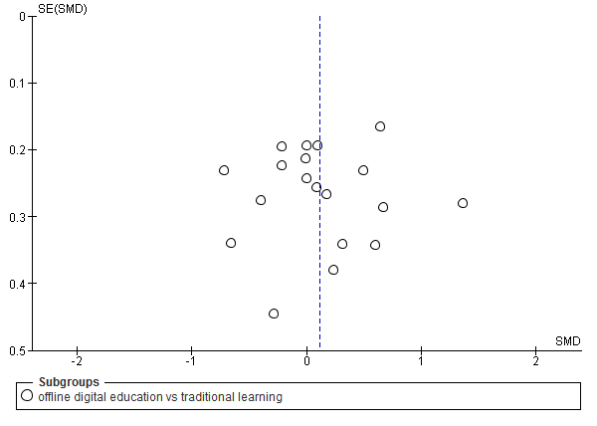
Funnel plot of studies comparing offline digital education with traditional, postintervention knowledge outcome. SMD: standardized mean difference.

As presented in Figure 5, the risk of bias was generally unclear or high in most of the studies because of a lack of relevant information in the included studies. For 14 (39%) studies, we found that the risk of bias was low in at least four of six domains [38,39,41,42,54,55,58,59,61,65-68]. For 22 studies (61%), we found that the risk of bias was high, as the studies had an unclear risk of bias in at least three of six domains or a high risk in at least one domain [10,35-37,40,43,44,45,46,47,48-53,56, 57,60,62-64]. A symmetrical funnel plot of studies comparing offline digital education and traditional learning suggests low risk of publication bias for the outcome knowledge (Figure 6). The overall risk of bias for cRCTs was unclear due to limited information from included studies (Multimedia Appendix 4).

## Discussion

### Principal Findings

Our findings show that offline digital education is as effective as traditional learning in improving medical students’ postintervention knowledge and may be more effective in improving skills, with effect sizes ranging from small (for knowledge) to large (for skills). We are uncertain about the effects for attitudes and satisfaction due to missing data or incomplete reporting. None of the studies reported on changes in accessibility or availability for education or adverse effects of the interventions. Only four studies reported the cost of offline digital education interventions; however, no estimates for comparator groups were provided.

Several limitations in the included literature need to be highlighted. For instance, we found that the evidence was of low quality due to the predominantly high risk of bias (studies’ limitations) or inconsistency (high heterogeneity of the pooled analyses). Furthermore, the included studies were highly heterogeneous in terms of student populations (years 1-5), comparator groups (traditional learning and different forms of offline digital education), outcomes and measurement tools (multiple-choice questionnaires, surveys, Likert-type scales, questionnaires, essays, quizzes, practical sections), study designs, settings (university or hospital), and interventions (learning contents, types of delivery mode, duration, frequency, intensity, and security arrangements). In addition, the duration frequency of the interventions were highly variable. Although the included studies encompass a reasonable range of interventions and content, the data are mostly limited to high-income countries, thereby limiting the generalizability of our findings to other settings including low- and middle-income countries.

We also found that reporting in the included studies was often poor. For example, four studies (11%) reported on the cost of setup of the interventions only (without any comparison data). Moreover, we found that none of the studies used learning theories underpinning the development or application of the offline digital education. Most of the studies (92%) used nonvalidated measurement instruments to quantify the outcomes, thereby jeopardizing the reliability and credibility of digital education research. Furthermore, 20 studies (56%) used software/computer programs as the main mode of delivery of the learning content. However, the technical aspects of these programs such as design or functions were often omitted from the studies.

Offline digital education has the potential to play an important role in medical students’ education, especially in low- and middle-income countries. Implementing offline digital education in medical education may require much less investment and infrastructure than alternative forms of digital education (eg, virtual reality or online computer-based education). Because of its scalability, offline digital education has the potential to reduce the shortage of medical doctors. It could be a major (for low- and middle-income countries) or an alternative (for high-income countries) mode of delivering education for medical schools across the world, as more than 4 billion people still did not have access to the internet as of 2016 [6].

To the best of our knowledge, there are only two reviews available in the literature that examined the effectiveness of offline digital education among similar populations [13,17]. One of these reviews, published in 2001, suggested that offline digital education (computer-assisted learning) could reduce the costs of education and increase the number of medical students [13]. However, no formal assessments on cost-related outcomes were made, and further research was recommended. A review by Rasmussen et al stated that offline digital education was equivalent or possibly superior to traditional learning in improving knowledge, skills, attitudes, and satisfactions of preregistration health professionals [17], which is largely in line with our findings. However, Rasmussen et al applied a much narrower search timeframe and focused on all preregistration health care professionals (ie, including students from medical, dental, nursing, and allied health care fields) and could not provide specific recommendations for medical students’ education. Our review provides up-to-date evidence with a comprehensive search strategy and a focus on medical students’ education and includes meta-analyses of studies for knowledge and skills.

### Strengths

Strengths of this systematic review include comprehensive searches with no language limitations, robust screening, independent data extractions, and risk-of-bias assessments. The review includes studies from the year 1990 in order to report the most comprehensive evidence and provides up-to-date evidence on the effectiveness of different types of offline digital education for medical students’ education.

### Limitations

Some limitations must be acknowledged while interpreting the results. First, we were unable to obtain missing information from the study authors despite multiple attempts. Second, we presented postintervention data rather than mean change scores, as the majority of the included studies (81%) reported postintervention data and only seven studies (19%) reported mean change scores. Third, we were unable to determine whether the study administrators received any incentives from the software or program developers, which might constitute bias. Lastly, we were unable to carry out prespecified subgroup analysis because of an insufficient number of studies under respective outcomes and because of the considerable heterogeneity of populations, interventions, comparators, and outcome measures used.

### Implications for Research and Practice

We believe that offline digital education interventions can be practically introduced in medical students’ education for improving their knowledge and skills in places where internet connectivity is limited, which may be of most concern in low- and middle-income countries. However, when interpreting the findings of this systematic review, stakeholders need to consider other factors such as students’ geographical location or features of the intervention such as interactivity, duration, frequency, intensity, and delivery mode.

Future studies should evaluate the cost-effectiveness, sustainability, and indirect (and direct) costs of the interventions (eg, time to develop or implement the educational module). Future research should also report on potential (or actual) adverse effects of the interventions. In addition, most of the studies assessed short-term effectiveness of the interventions; hence, there is a need to evaluate knowledge and skill retention during longer follow-ups (eg, 6-12 months). Additionally, other aspects of the interventions such as different levels of interactivity or feedback in low- and middle-income countries still need to be explored. Addressing these gaps in evidence will help policy makers and curriculum planners allocate resources appropriately.

### Conclusions

The findings from this review suggest that offline digital education is as effective as traditional learning in terms of medical students’ knowledge and may be more effective in improving their skills. However, the evidence on other outcomes is inconclusive or limited. Future research should evaluate the effectiveness of offline digital education interventions in low- and middle-income countries and report on outcomes such as attitudes, satisfaction, adverse effects, and economic impact.

## Appendix

Multimedia Appendix 1 MEDLINE (Ovid) Search Strategy.

Multimedia Appendix 2 Characteristics of the included studies.

Multimedia Appendix 3 Results of the included studies.

Multimedia Appendix 4 Risk of bias for cluster randomized controlled trials.

## Abbreviations

DVD: digital versatile disc
USB: universal serial bus
RCT: randomized control trial
cRCT: cluster randomized control trial
SMD: standardized mean difference
GRADE: Grading of Recommendations, Assessment, Development, and Evaluations
