# Supplementary material for: Offline Digital Education for Medical Students: Systematic Review and Meta-Analysis by the Digital Health Education Collaboration
Source: J Med Internet Res. 2019 Mar 25;21(3):e13165. doi: 10.2196/13165 (PMC6452290; doi:10.2196/13165)
Supplement: Multimedia Appendix 1 [file jmir_v21i3e13165_app1.pdf]

Multimedia Appendix 1: MEDLINE (Ovid) Search Strategy

1. exp education, professional/ not education, veterinary/

2. Education, Predental/

3. Education, Premedical/

4. exp Students, Health Occupations/

5. ((medic* or premedic* or dent* or laborator* or predent* or midwi?e* or nurs* or nutrition* or orthop* or podiat* or pharmac* or psycholog* or psychiatr* or health or healthcare or occupational therap* or physiotherap* or physical therap* or clinical or surg* or radiolog* or obstetric* or gyn?ecolog* or orthodont* or An?esthesi* or Dermatolog* or Oncolog* or Rheumatolog* or Neurolog* or Patholog* or P?ediatric* or Cardiolog* or Urolog*) adj3 (student* or graduate* or undergraduate* or staff or personnel or practitioner* or clerk* or fellow* or internship* or residen* or educat* or train* or novice* or tutor*)).tw,kf.

6. or/1-5

7. Computer-Assisted Instruction/

8. exp Internet/

9. Computer Simulation/

10. Patient Simulation/

11. software/

12. Mobile Applications/

13. User-Computer Interface/

14. Video Games/

15. Web Browser/

16. Education, Distance/

17. Computers/

18. exp Microcomputers/

19. exp Cell Phones/

20. Games, Experimental/

21. exp Models, Anatomic/

22. Audiovisual Aids/

23. Educational Technology/

24. Electronic Mail/

25. exp Telemedicine/

26. Telenursing/

27. Telecommunications/

28. Webcasts/

29. exp Videoconferencing/

30. ((computer* or digital* or hybrid or blended or mixed mode or distance or remote* or electronic or mobile or online* or interactiv* or multimedia or internet or web* or virtual* or game* or gaming or Videogame* or Videogaming) adj3 (classroom* or course* or educat* or instruct* or learn* or lecture* or simulat* or train* or teach* or tutor* or platform*)).tw,kf.

31. (Simulat* adj3 (course* or educat* or instruct* or learn* or train* or teach* or platform* or high-fidelity)).tw,kf.

32. e-learn*.tw,kf.

33. elearn*.tw,kf.

34. m-learn*.tw,kf.

35. mlearn*.tw,kf.

36. smartphone*.tw,kf.

37. smart-phone*.tw,kf.

38. ((mobile or cell) adj2 phone*).tw,kf.

39. iphone*.tw,kf.

40. android*.tw,kf.

41. ipad*.tw,kf.

42. Personal digital assistant*.tw,kf.

43. handheld computer*.tw,kf.

44. Mobile App?.tw,kf.

45. Mobile Application?.tw,kf.

46. webcast*.tw,kf.

47. webinar*.tw,kf.

48. flipped classroom*.tw,kf.

49. Serious game*.tw,kf.

50. Serious gaming.tw,kf.

51. Patient Simulat*.tw,kf.

52. Virtual patient*.tw,kf.

53. ((educat* or instruct* or learn* or simulat* or train* or teach* or interactiv*) adj2 technolog*).tw,kf.

54. Massive Open Online Course?.tw,kf.

55. Mooc?.tw,kf.

56. (Canvas network or Coursera or Coursesites or edx or Futurelearn or iversity or miriada x or moodle or novoed or openlearning or open2study or plato or spoc or udacity or pingpong).tw,kf.

57. or/7-56

58. 6 and 57

59. Education.fs.

60. Education/

61. Teaching/

62. Learning/

63. exp Inservice Training/

64. Curriculum/

65. educat*.tw,kf.

66. learn*.tw,kf.

67. train*.tw,kf.

68. instruct*.tw,kf.

69. teach*.tw,kf.

70. or/59-69

71. Health Personnel/

72. exp Allied Health Personnel/

73. Anatomists/

74. "Coroners and Medical Examiners"/

75. exp Dental Staff/

76. exp Dentists/

77. Health Educators/

78. Infection Control Practitioners/

79. Medical Laboratory Personnel/

80. exp Medical Staff/

81. exp Nurses/

82. exp Nursing Staff/

83. Personnel, Hospital/

84. Pharmacists/

85. exp Physicians/

86. Physician*.tw,kf.

87. Doctor*.tw,kf.

88. Nurs*.tw,kf.

89. Surg*.tw,kf.

90. Health Personnel.tw,kf.

91. healthcare professional*.tw,kf.

92. radiolog*.tw,kf.

93. dentist*.tw,kf.

94. Pharmacist*.tw,kf.

95. Hospital Administrator*.tw,kf.

96. Podiatr*.tw,kf.

97. Psycholog*.tw,kf.

98. Psychiatr*.tw,kf.

99. An?esthesi*.tw,kf.

100. Clinician*.tw,kf.

101. Dermatolog*.tw,kf.

102. General practioner*.tw,kf.

103. Cardiolog*.tw,kf.

104. Oncolog*.tw,kf.

105. Rheumatolog*.tw,kf.

106. Neurolog*.tw,kf.

107. Patholog*.tw,kf.

108. P?ediatric*.tw,kf.

109. Physiotherap*.tw,kf.

110. Physical therap*.tw,kf.

111. Occupational therap*.tw,kf.

112. dieti?ian*.tw,kf.

113. Dietetic*.tw,kf.

114. midwi?e*.tw,kf.

115. nutrition*.tw,kf.

116. orthopti*.tw,kf.

117. obstetric*.tw,kf.

118. gyn?ecolog*.tw,kf.

119. orthodont*.tw,kf.

120. Urolog*.tw,kf.

121. or/71-120

122. Health Occupations/

123. exp Allied Health Occupations/

124. Biomedical Engineering/

125. Chiropractic/

126. exp Dentistry/

127. exp Evidence-Based Practice/

128. exp Medicine/

129. exp Nursing/

130. Dietetics/

131. Optometry/

132. Orthoptics/

133. exp Pharmacology/

134. exp Pharmacy/

135. Podiatry/

136. Psychology, Medical/

137. Serology/

138. Specialization/

139. exp Surgical Procedures, Operative/

140. exp Radiography/

141. or/122-140

142. 121 or 141

143. 57 and 70 and 142

144. Psychomotor Performance/

145. motor skills/

146. ((psychomotor or procedural or technical) adj3 skill*).tw,kf.

147. (psychomotor adj3 performance).tw,kf.

148. or/144-147

149. 6 and 148

150. 58 or 143 or 149

151. limit 150 to yr="1990 -Current"

152. randomized controlled trial.pt.

153. controlled clinical trial.pt.

154. randomized.ti,ab.

155. placebo.ti,ab.

156. drug therapy.fs.

157. randomly.ti,ab.

158. trial.ti,ab.

159. groups.ti,ab.

International Clinical Trials Platform (ICTRP) – WHO

Source Link: http://apps.who.int/trialsearch/AdvSearch.aspx

Data sets from data providers are updated every Wednesday evening according to the following schedule:

Every week:

- Australian New Zealand Clinical Trials Registry, last data file imported on **16 August 2017**
- Chinese Clinical Trial Registry, last data file imported on **16 August 2017**
- ClinicalTrials.gov, last data file imported on **16 August 2017**
- EU Clinical Trials Register (EU-CTR), last data file imported on **16 August 2017**
- ISRCTN, last data file imported on **16 August 2017**
- The Netherlands National Trial Register, last data file imported on **16 August 2017**

Every 4 weeks:

- Brazilian Clinical Trials Registry (ReBec), last data file imported on **16 August 2017**
- Clinical Trials Registry - India, last data file imported on **16 August 2017**
- Clinical Research Information Service - Republic of Korea, last data file imported on **16 August 2017**

- Cuban Public Registry of Clinical Trials, last data file imported on **16 August 2017**
- German Clinical Trials Register, last data file imported on **16 August 2017**
- Iranian Registry of Clinical Trials, last data file imported on **16 August 2017**
- Japan Primary Registries Network, last data file imported on **16 August 2017**
- Pan African Clinical Trial Registry, last data file imported on **16 August 2017**
- Sri Lanka Clinical Trials Registry, last data file imported on **16 August 2017**
- Thai Clinical Trials Register (TCTR), last data file imported on **16 August 2017**
- ****New**** Peruvian Clinical Trials Registry (REPEC), last data file imported on **16 August 2017**

SEARCH 1

Using the title field in the advanced search, searching ALL registered trials

e-learning OR elearning OR m-learning OR mlearning OR "Canvas network" OR Coursera OR Coursesites OR edx OR Futurelearn OR iversity OR "miriada x" OR moodle OR novoed OR openlearning OR open2study OR plato OR spoc OR udacity OR pingpong OR "Massive Open Online Course*" OR Mooc OR Moocs OR smartphone* OR smart-phone* OR iphone* OR android* OR ipad* OR "personal digital assistant*" OR "handheld computer*" OR "mobile app" OR "mobile apps" OR "mobile application" OR "mobile applications" OR webcast* OR webinar* OR "flipped classroom*" OR "serious game*" OR "serious gaming" OR "patient simulat*" OR "virtual patient*" OR "psychomotor performance"

SEARCH 2

Using the title field in the advanced search, searching ALL registered trials

(comput* OR digital* OR hybrid OR blended OR "mixed mode" OR distance OR remote* OR electronic OR mobile OR online* OR interactiv* OR multimedia OR internet OR web* OR virtual OR game* OR gaming OR videogame* OR videogaming OR simulat* OR virtual OR technolog*)

AND

(classroom* OR course* OR educat* OR instruct* OR learn* OR lecture* OR simulat* OR train* OR teach* OR tutor* OR platform* OR "high-fidelity")
