# Supplementary material for: Offline Digital Education for Medical Students: Systematic Review and Meta-Analysis by the Digital Health Education Collaboration
Source: J Med Internet Res. 2019 Mar 25;21(3):e13165. doi: 10.2196/13165 (PMC6452290; doi:10.2196/13165)
Supplement: Multimedia Appendix 2 [file jmir_v21i3e13165_app2.pdf]

Multimedia Appendix 2: Characteristics of included studies.

Study ID (Author Year), Reference, Study Design, country	Population (N[a]), Medical student (year)	Setting, Source of Funding	Field of study	Intervention type	Duration of the intervention	Control	Outcomes
Ackermann 2010 [35], RCT[b], Germany	20, Not specified	University, NR[c]	Surgery (Orthopedic Surgery)	CD-ROM[d]	4-6 hours	Traditional learning	Skill
Amesse 2008 [36], RCT, USA	36, Third year	Hospital, NR	Radiology	CD-ROM	1.5 hours	Traditional learning	Knowledge
Armstrong 2009 [37], RCT, UK	21, Fourth year	Hospital, NR	Arterial Blood Gas Interpretation	PowerPoint Presentation	1 week	Traditional learning	1) Knowledge 2) Satisfaction
Carrero 2009 [38], RCT, Spain	68, Third year	University, NR	Basic Life Support Algorithms	PowerPoint Presentation	1 hour	Traditional learning	Knowledge
Cheng 2017 [39], RCT, USA	41, Second, Third and Fourth Year	University, NR	Orthopedics	Computer-based Video	10 minutes	Traditional learning	Skill
Davis 2008 [40], RCT, UK	229, First year	University, NR	Evidence based Medicine	CD-ROM	40 minutes	Traditional learning	1) Knowledge 2) Attitude
de Jong 2010 [41], RCT, The Netherlands	107, Third year	University, NR	Musculoskeletal problems	PowerPoint Presentation	3 weeks	Traditional learning	1) Knowledge 2) Satisfaction
Desch 1991 [42], RCT, USA	78, Third year	University, NR	Pediatrics (Neonatal Management)	Software Program	1 week	Traditional learning	1) Knowledge 2) Satisfaction 3) Cost
Devitt 1999 [43], Australia	90, Second year	University, NR	Anatomy and Physiology	Software Program	2 weeks	Software Program	Knowledge
Elves 1997 [44], RCT, UK	26, Third year	Hospital, IS[e]	Urology	Offline digital education (blended)	1 week	Traditional learning	1) Knowledge 2) Satisfaction
Fasce 1995 [45], RCT, Chile	100, Fourth year	University, NR	Medicine (Hypertension)	Software Program	5 hours	Traditional learning	1) Knowledge 2) Attitude 3) Satisfaction
Finley 1998 [46], RCT, Canada	40, Second year	University, NR	Medicine (Auscultation of Heart)	CD-ROM	3 days	Software Program	1) Knowledge 2) Satisfaction
Gelb 2001 [47], RCT, USA	107, Not specified	University, NIS[f]	Anatomy	Offline digital education (unspecified)	-	Traditional learning	1) Knowledge 2) Satisfaction
Green 2011 [48], RCT, USA	121, Second year	Hospital, NIS	Advanced Care Planning	Software Program	-	Traditional learning	1) Knowledge 2) Skill 3) Satisfaction
Hilger 1996 [49], RCT, USA	77, Third year	University, NR	Medicine (Pharyngitis)	Software Program	1 week	Traditional learning	1) Knowledge 2) Attitude
Hudson 2004 [10], RCT, Australia	100, Third year	University, NIS	Neuroanatomy and neurophysiology	Software Program	2 weeks	Software Program	Knowledge
Holt 2001 [50], RCT, UK	185, First year	University, NR	Endocrinology	Software Program	6 hours	Traditional learning	1) Knowledge 2) Satisfaction 3) Cost
Lee 1997 [51], RCT, USA	82, Second year	University, NR	Biochemistry/Acid-Base Problem Solving	Software Program	2.5 hours	Traditional learning	1) Knowledge 2) Satisfaction
MacFadyen 1993 [52], RCT, Canada	54, Fourth year	University, NIS	Clinical pharmacology	Offline digital education (unspecified)	12.2 hours (average)	Traditional learning	1) Knowledge 2) Attitude
Mangione 1991 [53], RCT, USA	35, Third year	University, NIS	Auscultation of the heart	Software Program	6 weeks	Traditional learning	1) Knowledge 2) Attitude
McDonough 2002 [54], RCT, UK	37, Third year	University, NR	Psychiatry	Software Program	1.5 hours	Traditional learning	1) Knowledge 2) Satisfaction
Mojtahedzadeh 2014 [55], RCT, Iran	61, Third year	University, NIS	Physiology of hematology and oncology	CD-ROM	2.5 hours	Traditional learning	1) Knowledge 2) Satisfaction

Nola 2005 [56], cRCT[g], Croatia	225, Not specified	University, NIS	Pathology	Software Program	1 term	Traditional learning	Knowledge
Perfeito 2008 [57], RCT, Brazil	35, Fourth year	University, NR	Surgery	CD-ROM	1.5 hours	Traditional learning	1) Knowledge 2) Satisfaction
Pusic 2007 [58], RCT, Canada and USA	152, Final year	University, NIS	Radiology	Software Program	1 hour	Software Program	1) Knowledge 2) Satisfaction
Ram 1997 [59], RCT, Malaysia	64, Final year	Hospital, NR	Cardiology	Software Program	1 hour	Traditional learning	Knowledge
Santer 1995 [60], RCT, USA	179, Third and Fourth Year	University, NR	Pediatrics	Software Program	1 hour	Traditional learning	1) Knowledge 2) Satisfaction
Seabra 2004 [61], RCT, Brazil	60, Second and third year	University, NR	Urology	Software Program	2 hours	Traditional learning	1) Knowledge 2) Satisfaction
Shomaker 2002 [62], RCT, USA	94, Second year	University, NR	Parasitology	Software Program	26.8 hours[h]	Traditional learning	1) Knowledge 2) Satisfaction
Solomon 2004 [63], RCT, USA	29, Third year	University, NIS	Learning concepts (Digital and Live Lecture formats)	CD-ROM	-	Traditional learning	Knowledge
Stanford 1994 [64], RCT, USA	175, First year	University, NIS	Anatomy (Cardiac Anatomy)	Software Program	47.91 minutes (average)	Traditional learning	1) Knowledge 2) Satisfaction
Summers 1999 [65], RCT, USA	69, First year	University, NIS	Surgery	Software Program	6.75 hours	Traditional learning	1) Knowledge 2) Skill
Taveira-Gomes 2015 [66], RCT, Portugal	96, Fourth and fifth year	University, NR	Cellular Biology	Software Program	1 hour	Software Program	Knowledge
Vichitvejpaisal 2001 [67], RCT, Thailand	80, Third year	University, NIS	Arterial Blood Gas Interpretation	Software Program	1 day	Traditional learning	Knowledge
Vivekananda-Schmidt 2005 [68], RCT, UK (Newcastle)	241, Third year	University, NIS	Orthopedics (Musculoskeletal Examination Skills)	CD-ROM	1 hour	Traditional learning	1) Skill 2) Cost
Vivekananda-Schmidt 2005a [68], RCT, UK (London)	113, Third year	University, NIS	Orthopedics (Musculoskeletal Examination Skills)	CD-ROM	1 hour	Traditional learning	1) Skill 2) Cost

Footnotes

[a]Number of participants, [b]randomized controlled trial, [c]Not reported , [d]Compact Disc Read Only Memory, [e]Industrial Sponsorship, [f]Non-industrial sponsorship, [g]cluster randomized controlled trial, [h]Average time
