# Supplementary material for: Offline Digital Education for Medical Students: Systematic Review and Meta-Analysis by the Digital Health Education Collaboration
Source: J Med Internet Res. 2019 Mar 25;21(3):e13165. doi: 10.2196/13165 (PMC6452290; doi:10.2196/13165)
Supplement: Multimedia Appendix 3 [file jmir_v21i3e13165_app3.pdf]

Multimedia Appendix 3: Results of the included studies.

**Primary outcomes**									
**Study ID, N[a]**	**Type(s) of comparison**	**Timing of outcome assessment (Postintervention and follow-up)**	**Intervention group**		**Control group**		**Between-group difference**		**Standardized Mean Difference (95% CI)**
			**Mean**	**Standard deviation**	**Mean**	**Standard deviation**	**Mean difference[b]**	**95% CI[b]**	
**Knowledge**									
Amesse et al 2008, 36	Offline digital education versus traditional learning	Postintervention	74	11	67	12	7	-0.52,14.52	0.59 (-0.07, 1.26)
Armstrong et al 2009, 21	Offline digital education versus traditional learning	Postintervention	68.8	10.54	73.3	17.75	-4.50	-16.68, 7.68	-0.29 (-1.15, 0.58)
Carrero et al 2009, 68	Offline digital education versus traditional learning	Postintervention	2.3	0.38	2.3	0.38	0.00	-0.18,0.18	0 (-0.48, 0.48)
Davis et al 2008, 229	Offline digital education versus traditional learning	Postintervention	0.8	3.2	1.3	2.4	-0.8	-1.4, -0.1	-0.18 (-0.48, 0.12)
de Jong et al 2010, 107	Offline digital education versus traditional learning	Postintervention	6.6	1	6.6	1.1	0.00	-0.40, 0.40	0 (-0.40, 0.40)
Desch et al 1991, 78	Offline digital education versus traditional learning	Postintervention	19.4	0.7	18.8	1.04	0.60	0.12, 1.08	0.67 (0.11, 1.23)
Devitt and Palmer 1999, 90	Offline digital education versus offline digital education	At 2 weeks postintervention	37.8	2.7	32.9	3.2	4.90	3.16, 6.64	1.62 (0.93, 2.31)
Elves et al 1997, 26	Blended digital education versus traditional learning	At 1 week postintervention	10.2	4.0	5.0	3.5	5.20	2.31, 8.09	1.34 (0.48, 2.20)
Fasce et al 1995, 100	Offline digital education versus traditional learning	Postintervention	71.4	-	64.6	-	-	-	-
Finley et al 1998, 40	Offline digital education versus offline digital education	At 3 days postintervention	-	-	-	-	-	-	-
Gelb 2001, 107	Offline digital education versus traditional learning	Postintervention, At 26 days postintervention	6.7	1.8	6.5	2.2	0.2	-0.56, 0.96	0.10 (-0.28, 0.48)
Green and Levi 2011, 121	Offline digital education versus traditional learning	Postintervention	88[c]	-	85[c]	-	-	-	-
Hilger et al 1996, 75	Offline digital education versus traditional learning	At 3-4 weeks postintervention	78.4	-	-	73.4	-	-	-

Holt et al 2001, 185	Offline digital education versus traditional learning	Postintervention	64.8	19.83	69.8	25.69	-5	-0.51, 0.08	-0.22 (-0.60, 0.16)
Hudson 2004, 100	Offline digital education versus offline digital education	Postintervention	66[d]	-	70[e]	-	-	-	-
Lee et al 1997, 82	Offline digital education versus traditional learning	Postintervention	12.3	1.64	12.7	1.98	-0.4	-1.18, 0.38	-0.22 (-0.65, 0.22)
MacFadyen et al 1993, 54	Offline digital education versus traditional learning	Postintervention	58.0	5.0	60.0	5.0	-2.00	-4.67, 0.67	-0.39 (-0.93, 0.15)
Mangione et al 1991, 35	Offline digital education versus traditional learning	At 6 weeks postintervention	-	-	-	-	-	-	-
McDonough and Marks 2002, 37	Offline digital education versus traditional learning	Postintervention	57.1	13.5	66.5	14.6	-9.40	-18.47, -0.33	-0.65 (-1.32, 0.01)
Mojtahedzadeh et al 2014, 61	Offline digital education versus traditional learning	Postintervention	13.7	1.1	13.5	1.2	0.20	-0.40, 0.80	0.17 (-0.35, 0.69)
Nola et al 2005, 225	Offline digital education versus traditional learning	Postintervention	81.9	9.8	73.3	14.2	8.60	5.13, 12.07	0.64 (0.32, 0.96)
Perfeito et al 2008, 35	Offline digital education versus traditional learning	Postintervention	7.1	0.87	6.8	1.02	0.31	-0.32, 0.94	0.31 (-0.36, 0.98)
Pusic et al 2007, 152	Offline digital education versus offline digital education	Postintervention	3.9	1.2	3.9	1.2	0.00	-0.33, 0.33	0.00 (-0.33, 0.33)
Ram 1997, 64	Offline digital education versus traditional learning	Postintervention	57.4	8.7	46.5	7.1	10.90	7.01, 14.79	1.36 (0.81, 1.90)
Santer et al 1995, 179	Offline digital education versus traditional learning	Postintervention, At 11-22 months follow-up	16.6<br>15.3	2.3<br>2.8	15.6<br>14.6	3.6<br>2.3	1<br>0.70	-0.34,2.34<br>-0.82,2.22	0.33 (-0.12, 0.77)<br>0.27 (-0.32, 0.86)
Seabra et al 2004, 60	Offline digital education versus traditional learning	Postintervention	15.1	-	15.6	-	-	-	-
Shomaker et al 2002, 94	Offline digital education versus traditional learning	Postintervention, At 4 months follow-up	88.7<br>57.5	6.46<br>19.48	88.1<br>52.5	6.91<br>12.84	0.60<br>1.70	-2.76, 3.96<br>-8.90, 12.30	0.09 (-0.41, 0.59)<br>0.30 (-0.20, 0.81)
Solomon et al 2004, 29	Offline digital education versus traditional learning	Postintervention	4.8	2	4.4	1.08	0.40	-0.73, 1.53	0.23 (-0.51, 0.97)
Stanford et al 1994, 175	Offline digital education versus traditional learning	Postintervention	41.5	18	41.7	13.6	-0.20	-6.87, 6.47	-0.01 (-0.43, 0.41)
Summers et al 1999, 69	Offline digital education versus traditional learning	Postintervention, At 1 month follow-up	49<br>-	-<br>-	63<br>-	-<br>-	-14<br>-	-<br>-	-<br>-

Taveira-Gomes et al 2015, 96	Offline digital education versus offline digital education	Postintervention,<br>At 1 week follow-up	72.7<br>82.3	18.3<br>15	33<br>42	18<br>20.7	39.70<br>40.30	32.51, 46.89<br>33.14, 47.46	2.17 (1.67, 2.67)<br>2.21 (1.71, 2.72)
Vichitvejpaisal et al 2001, 80	Offline digital education versus traditional learning	Postintervention,<br>At 3 weeks follow-up	57.8<br>48.1	10.74<br>8.54	65.8<br>50.0	11.32<br>9.71	-8.00<br>-1.90	-12.84, -3.16<br>-5.91, 2.11	-0.72 (-1.17, -0.27)<br>-0.21 (-0.65, 0.23)
**Skills**									
Ackermann et al 2010, 20	Offline digital education versus traditional learning	Postintervention	4.1	2.78	3.8	1.2	0.30	-1.58, 2.18	0.13 (-0.74, 1.01)
Cheng et al 2017, 41	Offline digital education versus traditional learning	Postintervention	5.5	1.1	1.6	1.1	3.90	[3.22, 4.58]	3.48 (2.48, 4.48)
Green and Levi 2011, 121	Offline digital education versus traditional learning	Postintervention	-	-	-	-	-	-	-
Summers et al 1999, 69	Offline digital education versus traditional learning	Postintervention,<br>At 1 month follow-up	390[f]<br>427[f]	-	385[g]<br>396[g]	-	-	-	-
Vivekananda-Schmidt et al 2005 (Newcastle), 241	Offline digital education versus traditional learning	Postintervention	20.5	3.2	18.8	3.2	1.70	0.69, 2.71	0.53 (0.21, 0.85)
Vivekananda-Schmidt 2005a et al (London), 113	Offline digital education versus traditional learning	At 1-10 months after completion of the module	15.1	1.8	14.1	2.4	1.00	0.20, 1.80	0.46 (0.07, 0.85)
**Attitude**									
Davis et al 2008, 229	Offline digital education versus traditional learning	Postintervention	-	-	-	-	-	-	-
Fasce et al 1995, 100	Offline digital education versus traditional learning	Postintervention	89.4	-	46.9	-	-	-	-
Hilger et al 1996, 75	Offline digital education versus traditional learning	Postintervention	-	-	-	-	-	-	-
Mangione et al 1991, 35	Offline digital education versus traditional learning	Postintervention	63.8	21.1	-	-	-	-	-
MacFadyen et al 1993, 54	Offline digital education versus traditional learning	Postintervention,<br>At 5 weeks follow-up	2.8<br>-	0.14<br>-	2.4<br>-	0.1<br>-	0.40<br>-	0.32, 0.48<br>-	2.71 (1.96, 3.47)<br>-
**Satisfaction**									
Desch et al 1991, 78	Offline digital education versus traditional learning	-	-	-	-	-	-	-	-
Elves et al 1997, 26	Blended digital education versus traditional learning	Postintervention	3	0.9	-	-	-	-	-

Fasce et al 1995, 100	Offline digital education versus traditional learning	Postintervention	89.4	-	98.1	-	-	-	-
Finley et al 1998, 40	Offline digital education versus offline digital education	-	-	-	-	-	-	-	-
Gelb 2001, 107	Offline digital education versus traditional learning	-	-	-	-	-	-	-	-
Green and Levi 2011, 121	Offline digital education versus traditional learning	Postintervention	7.8	-	5.6	-	-	-	-
McDonough and Marks 2002, 37	Offline digital education versus traditional learning	Postintervention	3.5	2	5.8	1.3	-2.30	-3.38, -1.22	-1.33 (-2.05, -0.61)
Lee et al 1997, 82	Offline digital education versus traditional learning	Postintervention	2.6	1.1	2.5	1.1	0.10	-0.38, 0.58	0.09 (-0.35, 0.53)
Mojtahedzadeh et al 2014, 61	Offline digital education versus traditional learning	Postintervention	1.93	-	2.31	-	-	-	-
Perfeito et al 2008, 35	Offline digital education versus traditional learning	-	-	-	-	-	-	-	-
Pusic et al 2007, 152	Offline digital education versus offline digital education	Postintervention	55[c]	-	81[c]	-	-	-	-
Santer et al 1995, 179	Offline digital education versus traditional learning		-	-	-	-	-	-	-
Seabra et al 2004, 60	Offline digital education versus traditional learning	-	-	-	-	-	-	-	-
Shomaker et al 2002, 94	Offline digital education versus traditional learning	-	-	-	-	-	-	-	-
Stanford et al 1994, 175	Offline digital education versus traditional learning	-	-	-	-	-	-	-	-
			**Intervention**		**Control**		**Risk Ratio**	**95% CI**	
			**Events**	**Total**	**Events**	**Total**	**-**	**-**	
Armstrong et al 2009, 21	Offline digital education versus traditional learning	Postintervention	9	9	10	12	1.18	0.87, 1.59	
de Jong et al 2010, 107	Offline digital education versus traditional learning	Postintervention	15	38	39	45	0.46	0.30, 0.69	
Holt et al 2001, 185	Offline digital education versus traditional learning	Postintervention	36	42	0	0	-	-	

Footnotes

[a]Number of participants, [b]Mean Differences and Confidence Intervals were calculated based on RevMan5.3 built-in function, [c]Percentage of the correct responses, [d]Didactic group, [e]Free text group, [f]Performance Quotient (PQ) score of computer-based training group, [g]PQ score of didactic training group, "-" = Not Available.
