# Supplementary material for: Offline Digital Education for Medical Students: Systematic Review and Meta-Analysis by the Digital Health Education Collaboration
Source: J Med Internet Res. 2019 Mar 25;21(3):e13165. doi: 10.2196/13165 (PMC6452290; doi:10.2196/13165)
Supplement: Multimedia Appendix 4 [file jmir_v21i3e13165_app4.pdf]

Multimedia Appendix 4: Risk of Bias for Cluster RCT

Study ID	Recruitment bias	Baseline imbalance	Loss of clusters	Incorrect analysis	Comparability with individual trials
Vivekananda-Schmidt 2005 (Newcastle)	Low	low	Unclear	Low	Unclear
	Recruitment of participants were carried out before randomization of clusters. It was mentioned in Figure 5 of the study.	No baseline difference was reported	Loss of cluster was not reported.	Analysis was carried out using a hierarchical linear regression with 2 levels (cluster level and student level). “Cluster” was entered as a random variable to account for the variance between individual clusters.	Comparison between cRCT and RCT could not be made due to heterogeneity in the specialization of participants and interventions.
Vivekananda-Schmidt 2005a (London)	Low	low	Unclear	Low	Unclear
	Recruitment of participants were carried out before randomization of clusters. It was mentioned in Figure 5 of the study.	No baseline difference was reported.	Loss of cluster was not reported.	Analysis was carried out using a hierarchical linear regression with 2 levels (cluster level and student level). “Cluster” was entered as a random variable to account for the variance between individual clusters.	Comparison between cRCT and RCT could not be made due to heterogeneity in the specialization of participants and interventions.
Nola 2005	Unclear	Unclear	High	Unclear	Unclear
	48 of the class of 225 students (20%) randomly enrolled in the computer-based teaching program and the remaining 80% of students of the same class were enrolled in the traditional teaching program used at the Zagreb University School of Medicine. Moreover, the author didn't report about cluster-randomization of the participants even though it mentioned the students were randomized in groups.	Baseline differences were not reported in this study.	Missing outcomes for individuals within clusters may lead to a risk of bias in cRCT.	No sufficient information to make judgement.	Comparison between cRCT and RCT could not be made due to heterogeneity in the specialization of participants and interventions.

Footnotes

RCT = Randomized controlled trial, cRCT = cluster RCT
